# Tasmanian Data Linkage Unit: Supporting innovative research, planning and policy formulation in Australia through the provision of high-quality linked-data services.

**DOI:** 10.23889/ijpds.v4i2.1137

**Published:** 2020-02-25

**Authors:** B Stokes, N Wiggins, T Albion, A Venn

**Affiliations:** 1 Menzies Institute for Medical Research, Medical Science Precinct 17 Liverpool Street Hobart TAS 7000 Australia

## Abstract

As a member of the Population Health Research Network Australia, being an Australian collaboration established to support the use of linked data for research and other purposes, the Tasmanian Data Linkage Unit [TDLU) provides linked-data services in Australia’s smallest state, and as part of the Menzies Institute for Medical Research at the state’s only University, the University of Tasmania. The TDLU works in close collaboration with the Tasmanian Government Department of Health and other key stakeholders both in Tasmania and Australia representing government, education, research, and the community sector. The TDLU is one of the newest data linkage services in Australia, and the smallest node of the PHRN having operated for almost nine years by less than three full time equivalent staff. However, despite its size and relative maturity as a provider of linked-data services, the TDLU continues to grow the number of datasets linked on a routine and ad-hoc basis, the number of projects completed, the size of its Master Linkage Map and number of ‘keys’ stored in this *Map*. The TDLU places high-emphasis on security, privacy preservation, innovation, quality assurance, stakeholder engagement and providing responsive and exemplary services to users of linked-data.

## Population Setting

Australia comprises six states and two territories, with Tasmania being the smallest state both in terms of land mass and population. Tasmania is an island located 240 kilometres to the south of the Australian mainland, comprising 68,401 square kilometres and representing less than 1% of the total area of Australia [1]. As at June 2017, the official population of Tasmania was 520,900 [2] persons with approximately 40% of persons residing in the greater metropolitan area of the state capital, Hobart. The 2016 Census of Population and Housing, released by the Australian Bureau of Statistics (ABS), show that indigenous Australians, being persons of Aboriginal and Torres Strait Islander origin, represented 2.8 per cent of the Australian population as counted in the 2016 Census [3]. In Tasmania, 4.4% of the total population identified as being of Aboriginal and Torres Strait Islander origin in this same census.

Tasmania is considered to have both a stable and ageing population, and regularly reports the slowest rate of population growth in Australia, with the broad consensus being that population growth is directly related to economic conditions [4]. Improved economic growth in recent years, however, has resulted in population growth currently exceeding long term annual trends. As at June 2017, the median age of the Tasmanian population was 42.2 years, being the highest of all Australian states and territories [5]. Tasmania also reported the largest increase in median age between 2012 and 2017, with an increase of 1.4 years [5]. The median age of males in 2017 was 41.0 years compared with 43.3 years for females. In greater Hobart, the median age of the population was 39.8 years as at June 2017, being a small increase from 39.2 in June 2012. The median age for the rest of Tasmania increased from 42.0 to 44.1 years over the same period [5]. 

## Operating Model

The Tasmanian Data Linkage Unit (TDLU) is a node of the Population Health Research Network (PHRN), being a collaboration established in 2009 as an initiative of the Australian Government, and through funding made available under a scheme known as the National Collaborative Research Infrastructure Strategy (NCRIS). In addition to commonwealth funding, state and territory governments and academic institutions make significant ongoing cash and in-kind contributions to support the development of linked-data services in Australia. In Tasmania, funding to operate the TDLU is provided to the state government Department of Health (DoH) under a formal agreement administered on behalf of the Commonwealth of Australia funding body by the University of Western Australia (UWA). In turn, the DoH contracts the University of Tasmania to operate the service with the only other funding to operate the TDLU coming from charges levied for linked-data services. Organisationally, the TDLU is located as part of the Menzies Institute for Medical Research (Menzies) in Hobart, which is an institute of the University and existing to perform internationally significant medical research leading to healthier, longer and better lives for Tasmanians. The TDLU commenced operation in 2010 and has operated continually since this time as part of the University. The TDLU receives significant in-kind support, both from the University and through the generous support of data custodians and related stakeholders.

## Governance

Having an effective governance model is of high importance to the TDLU. Strong governance supports a primary objective of the service, by directly contributing to the promotion, protection and maintenance of the wellbeing, health and prosperity of the population of Tasmania and Australia. A Management Committee comprising representatives of state and commonwealth government agencies and the community sector and tertiary education, meet three times annually to provide advice, including making recommendations regarding strategy, policy, funding priorities, stakeholder engagement, performance and accountability. The Tasmanian state government departments of Health, Education, Premier and Cabinet and Justice are represented on the Committee, together with the Australian Bureau of Statistics as the independent statistical agency of the Government of Australia. The *Committee* operates under agreed terms of reference, with minutes recorded and actions arising actively managed. A senior representative of the Tasmanian Government DoH is the nominated chair of the *Committee*, and has responsibility for convening and conducting meetings in accordance with its terms of reference. Members of the *Committee* bring a wealth of experience and expertise to support the operations of the TDLU in numerous areas including research, ethics, policy, planning, education, legal and technical. 

## Staffing

The TDLU is currently staffed by 2.8 full time equivalent (FTE) employees, with casual staff employed to perform clerical review services on an as required basis. At a high-level, staff responsibilities are broadly aligned across functions covering management, technical services, data-linkage functions and client support. As a small service, however, staff of the unit are required to be multi-skilled and work across a number of areas including management and planning, developing and maintaining stakeholder relationships, reporting, technical services, client servicing, quality assurance and administration. Employees of the service bring to the unit many years of experience working with administrative data at state and commonwealth levels. In addition to paid employees of the service, an experienced researcher and senior academic provides in-kind support as Director of the TDLU. This important role provides high-level direction to the service through extensive experience gained as a researcher using linked-data, and as a senior member of the UTAS leadership team that enables alignment of the role and function of the TDLU with the long-term vision of the UTAS and its many partners.

## Infrastructure

The TDLU’s linkage infrastructure is purposely designed to support high-quality research and evaluation, strategic planning and policy development to advance its mission in providing high-quality, and timely data linkage services. The MLM comprises over 1.8 million Master Linkage Keys (MLKs) representing individuals in the population with pointers to source records in health, education and other datasets. Applicants for linked data must meet strict criteria including submitting a formal application to the TDLU, providing all relevant current Human Research Ethics Committee (HREC) approvals from authorised entities, and obtaining the support of all data custodians involved in the research. This latter approval is sought in addition to HREC approvals, and prior to linkage work commencing.

Researchers are also required to provide comprehensive details on how data are proposed to be used, stored and destroyed at the end of the approved research. The TDLU receives applications for linked data from local and interstate researchers, with some applications made online using an application system developed by the PHRN. In such cases, it can be necessary to obtain HREC approvals from one or more additional ethics committees in other Australian jurisdictions. The TDLU complies with relevant privacy legislation and research guidelines issued by the National Health and Medical Research Council (NHMRC). These include the National Statement on Ethical Conduct in Human Research (2007, updated 2018) and that maximises conservation of individual privacy. The TDLU applies a range of operating procedures, supported by policies, that minimise release of linkage keys that could identify an individual in the population. 

## Physical Environment

The TDLU has in place, strict protocols, policies and procedures to maximise the safe storage of data provided for the purpose of creating and maintaining its MLM. Such protocols include a requirement to have card-controlled access for approved staff to a highly-secure operating facility, a separate secure, card-controlled server room that houses computing hardware, physical restrictions including network segmentation and electronic controls specific to environments, and hardware and software controls. The TDLU’s server infrastructure has no Internet or remote access capability; all custodian data and linkage keys are received and distributed from computers located on a separate physical local area network using a secure encrypted email service.

## Hardware

The TDLU utilises ‘industry-standard’, high-performance computing hardware based around Intel processors, that are readily supported by UTAS information technology staff. A separate database server, storage area network and backup server combine to provide the core components of its hardware infrastructure which are all housed in locked cabinets within secure server rooms. All hardware is replaced in line with broader UTAS hardware support policies, and are supported by formal vendor maintenance agreements. All TDLU hardware utilises Microsoft operating systems. The MLM and associated data are stored in a Microsoft SQL Server database.

## Software

At the core of the TDLU’s software infrastructure is a powerful commercial record linkage application that enables matching of data for individuals across disparate data sources. The application uses probabilistic data matching techniques to link together records for the same individual across datasets. Mathematical probabilities derived from a large reference dataset of known ‘matches’ and ‘non-matches’ are used to derive ‘match weights’ for each field. Separate weights are derived for field agreements, disagreements and missing values, with match weights being higher for variables with greater specificity (i.e., family name), and lower for variables with less specificity (i.e. gender). The weights are calculated from the logarithm of the frequency ratio of the field examined as shown in the diagram at Appendix A.

During the matching process, the agreement or disagreement weight for each field is added together to produce a combined score that represents the probability that matched records refer to the same individual in the population. The TDLU has an agreed threshold value above which a pair is considered a match, and another threshold below which it is considered not to be a match - threshold values are determined on a project by project basis. Between the two thresholds, a pair is considered to be a *‘possible match’*, and requires manual review by a Clerical Review Officer (CRO). The CRO uses specialised software developed by a staff member of the TDLU to aid the process of effectively determining ‘true’, ‘possible’, and ‘non-matches’. All linked data is stored in a single MLM consisting of individual MLKs. In addition to storing MLKs, the TDLU’s linkage infrastructure stores all source custodian data, maintains a full history of all links made and subsequent changes, and features custom built software to support quality control and clerical review functionality. A further custom designed software module generates spatial coordinates at a unit record level, that are primarily used to generate statistical area codes according to the Australian Statistical Geography Standard (2011) [6] . A comprehensive suite of Structured Query Language (SQL) queries are applied to linked datasets pre and post linkage to support standardisation of data prior to linkage and to improve overall quality of linkage keys.

## Security

The TDLU operates within a robust physical information security framework designed to offer the highest level of protection to data involved in linkage and research activities undertaken by the unit. The TDLU’s linkage infrastructure supports the creation of de-identified datasets, being researchable datasets with all identifying information such as name and address removed. Unique project person identifiers (linkage keys) are generated for each linkage project to ensure that data is not re-identifiable between research projects, with the ‘linkage key’ used to identify an individual in the population across datasets. De-identified researchable datasets directly assist users in universities, research institutes, government agencies and other organisations achieve a number of outcomes including being able to access information from a range of health and associated research datasets that would not otherwise be accessible, and to access ad-hoc survey data. The TDLU Security Framework is directed at ensuring that the TDLU establishes and maintains infrastructure to effectively and securely store data within a model of ‘best practice’ for a service of this kind, whilst protecting the privacy of personal information. Importantly, this framework is designed to be compliant with relevant UTAS policies and procedures, plus applicable Tasmanian laws and statutory obligations.

## Linkage Methodology

The TDLU utilises probabilistic data linkage techniques to support the construction and maintenance of its MLM. Probabilistic record linkage assumes there is no common identifier within the source databases being linked that can identify an individual in the population with complete accuracy. Probabilistic record linkage calculates the probability that two records belong to the same individual using multiple pieces of identifying information including title, first name, other names, surname, gender, date of birth, date of death and address. A record identifier is always provided by the data custodian, enabling the return of linkage keys so that clinical, service or activity data can be attached to the final de-identified, researchable dataset. In Tasmania, the public health system utilises a common, unique nine digit patient health identifier for health services provided in public hospitals, emergency departments and some non-admitted care settings that greatly improves linkage quality for datasets utilising this identifier.

In preparing datasets to be linked, and to establish a platform to achieve optimal links, the TDLU applies a series of data standardisation and cleaning strategies in advance of loading data into its data linkage engine. The linkage software engine further standardises data as a way of optimising the overall linkage process, to improve the quality of links generated, and to minimise the amount of clerical review required. Once data is standardised and loaded for linkage, the software engine undertakes deterministic linkage to identify obvious ‘true’ matches, and to reduce software processing time. Blocking techniques are applied to the linkage process as a way of stratifying the data and reducing the number of comparisons to be undertaken. Blocking variables aim to reduce search parameters across two datasets by avoiding comparing record pairs that are least likely to be matches. Without using blocking, every record in each dataset would need to be compared with every other record in the second data set. This would result in extremely large numbers of comparisons being required, which in turn would place high-demands on computing infrastructure, increase clerical review and significantly increase time required to perform linkage.

The TDLU applies a strict ‘model of separation’ in providing linked-data services. The separation principle [7] is a mechanism designed to protect the identity of individuals in a population and was developed to maintain privacy and allow data custodians to retain full control over access to their datasets. The separation principle is considered world’s best practice, and is widely used by PHRN supported data linkage centres in Australia. The ‘principle’ consists of four distinct steps, being that linkage units create linkage keys using a defined set of demographic variables, linkage staff extract and encrypt unique project-specific linkage keys for each project, encrypted linkage keys are released to relevant data custodians where approved clinical, activity or service variables are extracted, and finally the end-user receives a dataset from each data custodian using the unique ‘key’ to construct a researchable dataset. The separation principle ensures that no single party involved in record linkage (data custodian, data linkage unit and user of a linked dataset) can see all demographic or service information used to identify records relating to the same individual.

## Privacy and Data Sharing Legislation

The TDLU links custodian data through individual data exchange agreements, and without the explicit consent of individuals. Custodians collect data in accordance with relevant state and commonwealth privacy legislation specific to their operations and that underpin the collection, management and disclosure of data they hold. In Tasmania, individual privacy is regulated by the Right to Information Act 2009, and the Personal Information Protection Act 2004 with other more specific legislation applying to certain organisations including law enforcement. The Australian Government is currently reviewing commonwealth data sharing legislation in view of opportunities presented to increase the sharing of public data to support increased economic activity and productivity. 

## Datasets

In establishing the TDLU, significant focus was placed on establishing a formal Data Exchange Agreement (DEA) with the Tasmanian Government DoH, in their capacity as provider of publicly funded acute, sub-acute, non-acute and community health services. This agreement established a formal framework through which data collected for the purpose of providing healthcare services can be made available to the TDLU for constructing and maintaining its MLM to support approved human research projects. The agreement establishes the form and frequency of data provision, together with an agreed set of linkage variables. Public hospital admitted patient episodes, emergency department presentations, perinatal events, cancer notifications and community mental health episodes of care are major public health datasets routinely linked by the TDLU. In addition, births and deaths data are important datasets that are routinely linked, with exchange of data supported by a DEA with the data custodian.

Other state and commonwealth datasets are also linked, either routinely or ad-hoc, and are supported by enduring or specific one-off agreements. The TDLU maintains a close working relationship with all data custodians, both in obtaining data for linkage and in releasing linkage-keys so that approved variables can be attached to the linkage keys. Since inception, the TDLU has linked in excess of 65 individual datasets, representing over 8 million unit records and comprising approximately 1.8 million individuals in the Tasmanian and Australian population. Table 1 below shows the core datasets routinely linked by the TDLU, together with available date ranges, total records and unique keys produced.

**Summary of core datasets linked in TDLU’s MLM, date ranges, total records and unique keys:** 

Dataset	Date Range	Total Records	Unique Keys
Public Hospital Admitted Patient Episodes	2007 –2017	1,356,100	329,800
Public Hospital Emergency Department Presentations	2000 –2017	2,234,000	543,500
Deaths Registry Tasmania	1970 –2018	186,400	186,100
Births Registry Tasmania	2000 –2018	113,900	113,500
Tasmanian Cancer Registry	1982 –2016	102,400	88,400
Perinatal Data Collection – Mother (Public & Private)	2005 –2015	67,300	42,200
Perinatal Data Collection – Child (Public & Private)	2005 –2014	61,600	61,600
Australian Early Development Census (AEDC)	2009, 2012, 2015	855,800	855,000
Ambulance Tasmania Emergency Incidents	2010 –2015	321,500	151,000
Tasmanian Community Mental Health Data Collection	2000 - 2015	1,375,100	35,400

## Master Linkage Keys

The TDLU has developed a cross tabulated report that has been effective for a variety of purposes including providing potential applicants for linked-data with a visual representation of links existing between datasets. The report has also been an effective way of demonstrating to stakeholder groups continual growth in the number of keys stored in the TDLU’s MLM, and how many times individuals in the population receive services at multiple care settings including emergency departments, as inpatients in public hospitals and when transported to hospital by ambulance. A high-level summary of links stored in the TDLU’s MLM, specific to core datasets routinely linked, is shown in table 2.

**Unique keys stored in TDLU’s MLM by core dataset, links between datasets:** 

Dataset	No other file type	Ambulance	Admitted Paitent	Births	Cancer	Deaths	Emergency Department	Community Mental Health	Perinatal Baby Public Hospital	Perinatal Mother Public Hospital
Ambulance	13,300	151,000	108,000	12,400	20,400	28,500	132,600	15,100	7,600	8,500
Admitted Patient	17,600	108,000	329,800	63,300	38,300	42,900	279,800	23,400	38,100	29,800
Births	11,100	12,400	63,300	113,500	100	500	71,800	1,700	46,700
Cancer	9,900	20,400	38,300	100	88,400	53,400	46,800	2,400	100	500
Deaths	91,500	28,500	42,900	500	53,400	186,100	64,700	5,300	300	100
Emergency Department	155,000	132,600	279,800	71,800	46,800	64,700	543,500	30,500	34,600	26,300
Commuity Mental Health	3,200	15,100	23,400	1,700	2,400	5,300	30,500	35,400	500	3,100
Perinatal Baby Public Hospital	300	7,600	38,100	46,700	100	300	34,600	500	47,700
Perinatal Mother Public Hospital	1,100	8,500	29,800		500	100	26,300	3,100		33,100

Table 2 shows that a total of 543,500 individual keys are stored in the TDLU’s MLM for the emergency department dataset, with each key representing an individual in the population. Of these records, 155,000 individuals have no other record in any other dataset, 132,600 also have at least one ambulance record, 279,800 at least one public hospital admitted patient episode, 71,800 a birth record, 46,800 at least one cancer record, 64,700 a death record, 30,500 at least one community mental health record, 34,600 a public hospital perinatal event for the child and 26,300 a public hospital perinatal event for the mother.

### Applying For Linked Data

Researchers, policy makers, students, planners and other users of linked data are required to follow a set series of steps when applying for linked data from the TDLU. The high-level steps are:

1. Complete a TDLU Application for Linked Data
2. Obtain all relevant approvals (Human Research Ethics Committee, data custodians)
3. Data linkage and extraction
4. Review

Prior to submitting an application for linked-data, the TDLU recommends potential applicants communicate with staff of the TDLU to discuss the feasibility of their proposed project, approximate timeframes and pricing. Timeframes from applying for linked-data until obtaining datasets from custodians can vary significantly and are impacted by many factors including time taken to obtain all relevant approvals including custodian support, complexity of the project, current workload of the linkage unit, and the amount of clerical review and quality assurance required. All persons listed on HREC applications, and who will access the final researchable dataset, are required to meet set criteria including declaring any conflict of interest and signing a deed of agreement defining how the data will be used, stored and ultimately destroyed.

## Case study

The complexity of linkage projects performed by the TDLU has increased over time in line with a growing maturity of both the entity and end-users of linked-data services. Complexity has increased in areas including the total number of datasets linked, the number of records linked, the quality of source datasets for linkage, time periods of different datasets, cohort selection techniques, and linkage design to support multiple key extractions over time. For a small data-linkage unit with significant resource restrictions, complex projects present problems that constantly require innovative technological and operational solutions to ensure the timely processing of linkage projects that meet end-user expectations.

During 2018, the TDLU completed its most ambitious linkage project involving 14 disparate datasets and using three discrete cohort selections. The project, *‘Pathways To Better Health and Education Outcomes For Tasmania’s Children’*[8], involved a cohort of approximately 24,000 children in the Tasmanian population who interacted with one or more publicly provided services including perinatal health, early childhood services, child and parental health, early childhood centres, schools and health facilities. The research project is designed to provide practical analysis and insights into service provision and the role of Tasmania’s early childhood health and education services in supporting families and young children. The project is a partnership between researchers and multiple Tasmanian Government departments, together with the Telethon Kids Institute and the University of Western Australia, the Menzies and the School of Social Sciences at the University of Tasmania. Funding for the project is provided through a National Health and Medical Research Council (NHMRC) Partnership Project Grant and the Government of Tasmania partner organisations, with findings intended to provide outcomes-linked evidence required to inform future early childhood service planning from pregnancy to the start of full-time school.

For the TDLU, the project presented many challenges with linkage design being foremost and requiring the formulation of a complex design methodology, and extensive consultation with all project partners. Managing stakeholder expectations, obtaining all relevant custodian approvals, coordinating the supply of data from multiple custodians, planning the work to meet researcher timelines and within competing priorities, and clerically reviewing such a large project were just some of the challenges presented. At the conclusion of the linkage process, a comprehensive data quality statement was produced, being a feature of all linkage projects undertaken by the TDLU. For this project, the report contained a comprehensive array of metadata and linkage metrics, and described in detail the quality of each dataset linked and the steps taken to complete the linkage.

## Looking Forward

The TDLU has developed slowly, but consistently and methodically in the nine years it has been operating. The first 2.5 years were spent establishing strong foundations from which to establish enduring, robust, reliable and efficient linked-data services within a framework of privacy and with an unwavering focus on quality in all aspects of its operation. The time taken to establish data exchange agreements and infrastructure, develop policies and procedures, recruit skilled staff, put in place technological solutions, establish strong governance and market and promote services cannot be understated. Similarly, the time taken for results of linked-data projects to impact service or program delivery and/or directly impact research discoveries, can also be lengthy. However, through strong planning, perseverance and support to the small team of professionals by senior UTAS staff, the TDLU has successfully established a relatively small, but high-performing data linkage service with foundations enabling continued growth in the years to come.

## Ethics statement

The Tasmanian Data Linkage Unit operates in accordance with ethics approval provided by the Health and Medical Human Research Ethics Committee of the University of Tasmania.

## Appendix A: Linkage Weight Calculation

$$\text{Weight}=\log_{2}\left[\frac{\text{Frequency of agreement in LINKED pairs}}{\text{Frequency of agreement in UNLINKED pairs}}\right]$$

## References

[1] Australian Bureau of Statistics. 1384.6 Statistics Tasmania Canberra: ABS; 2008 [Available from: https://www.abs.gov.au/ausstats/abs@.nsf/0/D1C2967E40D51C1DCA2573C5000D9EC3?opendocument.

[2] Australian Bureau of Statistics. 3101.0 - Australian Demographic Statistics, Jun 2017 Canberra 2017 [Available from: https://www.abs.gov.au/ausstats/abs@.nsf/Previousproducts/3101.0Main%20Features2Jun%202017.

[3] Australian Bureau of Statistics. 2016 Census shows growing Aboriginal and Torres Strait Islander population Canberra: ABS; 2017 [Available from: https://www.abs.gov.au/ausstats/abs@.nsf/MediaRealesesByCatalogue/02D50FAA9987D6B7CA25814800087E03.

[4] Denny L. Tasmania’s Population Challenge: 650,000 by 2050 Hobart: Tasmanian Department of State Growth; 2015.

[5] Australian Bureau of Statistics. 3235.0 - Regional Population by Age and Sex, Australia Canberra: ABS; 2018 [Available from: https://www.abs.gov.au/AUSSTATS/abs@.Nsf/7d12b0f6763c78caca257061001cc588/cdd56c03c2fa5655ca2573210018d999!OpenDocument.

[6] Australian Bureau of Statistics. Australian Statistical Geography Standard (ASGS) Canberra: ABS; 2018 [Available from: https://www.abs.gov.au/websitedbs/D3310114.nsf/home/Australian+Statistical+Geography+Standard+(ASGS).

[7] Kelman CW, Bass AJ. Research use of linked health data - a best practice protocol. Australian and New Zealand journal of public health. 2002;26(3):251-5. 10.1111/j.1467-842x.2002.tb00682.x 12141621

[8] Telethon Kids Institute. Tassie Kids: A picture of early childhood services in Tasmania from birth to age five: Telethon Kids Institute; 2019 [Available from: https://www.telethonkids.org.au/projects/tassiekids/.

